# Technical considerations for evaluating clinical prediction indices: a case study for predicting code blue events with MEWS

**DOI:** 10.1088/1361-6579/abfbb9

**Published:** 2021-06-17

**Authors:** Kais Gadhoumi, Alex Beltran, Christopher G Scully, Ran Xiao, David O Nahmias, Xiao Hu

**Affiliations:** 1 School of Nursing, Duke University, Durham, NC, United States of America; 2 Department of Radiology and Biomedical Imaging, University of California San Francisco, San Francisco, CA, United States of America; 3 Office of Science and Engineering Laboratories, Center for Devices and Radiological Health, Food and Drug Administration, Silver Spring, MD, United States of America

**Keywords:** early warning score, clinical deterioration, predictive value of tests, vital signs, clinical alarms, performance evaluation

## Abstract

*Objective.* There have been many efforts to develop tools predictive of health deterioration in hospitalized patients, but comprehensive evaluation of their predictive ability is often lacking to guide implementation in clinical practice. In this work, we propose new techniques and metrics for evaluating the performance of predictive alert algorithms and illustrate the advantage of capturing the timeliness and the clinical burden of alerts through the example of the modified early warning score (MEWS) applied to the prediction of in-hospital code blue events. *Approach*. Different implementations of MEWS were calculated from available physiological parameter measurements collected from the electronic health records of ICU adult patients. The performance of MEWS was evaluated using conventional and a set of non-conventional metrics and approaches that take into account the timeliness and practicality of alarms as well as the false alarm burden. *Main results*. MEWS calculated using the worst-case measurement (i.e. values scoring 3 points in the MEWS definition) over 2 h intervals significantly reduced the false alarm rate by over 50% (from 0.19/h to 0.08/h) while maintaining similar sensitivity levels as MEWS calculated from raw measurements (∼80%). By considering a prediction horizon of 12 h preceding a code blue event, a significant improvement in the specificity (∼60%), the precision (∼155%), and the work-up to detection ratio (∼50%) could be achieved, at the cost of a relatively marginal decrease in sensitivity (∼10%). *Significance*. Performance aspects pertaining to the timeliness and burden of alarms can aid in understanding the potential utility of a predictive alarm algorithm in clinical settings.

## 1. Introduction

Early warning systems (EWS) are tools that warn about physiological instabilities in patients at risk of deterioration. They can play a crucial role in healthcare by enabling early and rapid intervention to help prevent in-hospital all-cause catastrophic events. Emerging tools are based on complex algorithms that use statistical and machine learning techniques to identify precursors of adverse events in single or multimodal physiological variables available at the bedside. However, the lack of comprehensive validation of many such tools to inform their implementations is one of the reasons that are responsible for their limited adoption in clinical practice (Damen *et al* 2016, Linnen *et al* 2019).

Methodological validation of EWSs is key to understanding their performance to predict clinical events. Conventional metrics typically used to evaluate the performance of EWSs are often limited to measures of sensitivity, specificity and discriminability (through concordance statistics), and often they are not evaluated in a way that considers the dynamic nature of a score from continuous vital sign data. These metrics are useful to evaluate the classification power of an EWS but may not describe important aspects of an EWS. Questions about the frequency of high scores and the burden from score-based alarms, and about the extent, the pattern, and the time frame over which EWS changes are observed before an adverse event cannot be answered by examining conventional metrics alone. For example, an EWS that often warns about an impending cardiac arrest too early (e.g. a week) or too late (e.g. seconds) may have limited clinical utility. Yet, depending on the study design, such warnings could be considered true predictions. Accuracy and timeliness of EWS changes are important characteristics of performance to help understand the potential clinical utility.

In this study, we consider performance metrics for clinical prediction indices and examine the types of information they can provide. To focus on developing the approaches for validating EWS that are agnostic to algorithms driving EWS, we conduct a case study where a simple modified early warning score (MEWS) was implemented to predict code blue events in ICU patients. MEWS was proposed as a screening tool to identify inpatients at risk for deterioration and to trigger early evaluation and transfer to step-down or intensive care (Subbe *et al* 2001). Views on the clinical usefulness of MEWS remain rather controversial despite its common use (Gao *et al* 2007, Alam *et al* 2014). The goal of this study is not to prove or disprove the predictive power of MEWS in ICU patients, since MEWS was designed and has generally been evaluated for identifying patient deterioration in broad hospital populations and not a predictive score for cardiac arrest in the ICU (Morgan and Wright 2007). Rather, our goal is to explore analysis methods for these types of scores, and we use MEWS as an example due to its simplicity to calculate and frequent appearance in research studies evaluating its performance.

## 2. Materials and methods

### 2.1. Data

Demographics and vital signs measurements were extracted from the electronic health record (EHR) of patients hospitalized between 1 March, 2013 and 31 December, 2017 at the University of California San Francisco (UCSF) Medical Center. Patients aged 18 years and older who were admitted in the intensive care unit (ICU) without a do-not-resuscitate order were included. For each patient, we collected the age, gender, and measurements of heart rate (HR), systolic blood pressure (SBP), respiratory rate (RR), temperature (Temp), and Glasgow Coma Score (GCS). The study was approved for research investigation by the UCSF institutional review board.

Patients were split into case and control groups. Case patients (*n* = 283 of 3410; 8.3%) were defined as those with at least one in-hospital code blue event, including cardiopulmonary arrest (182 cases), acute respiratory compromise (34 cases) and other medical emergencies (67 cases), as documented and confirmed by the code blue committee of the medical center. In order to control for any increased risk of another code blue event following a first occurrence, only data recorded between the time of ICU admission and the time of the first code blue event were retained for analysis. Control patients (*n* = 3127 of 3410; 91.7%) were defined as those who did not experience a code blue event during their stay. Data recorded between the time of ICU admission and discharge of control patients were extracted for analysis. The median length of ICU stay was 86.3 h in the case group (IQR = 245.9 h) and 160.9 h in the control group (IQR = 193.2 h).

Vital signs were available on an irregular time interval. On average, a new vital sign (HR, SBP, RR, Temp) was measured every 0.6 h in the case group and every 1.7 h in the control group. GCS was less frequently measured than vital signs.

### 2.2. MEWS calculation

MEWS is derived using a set of point assignment rules applied to physiological parameter measurements as shown in table 1 (Subbe *et al* 2001) (some institutions may implement modified versions of these rules and/or use other/different physiological parameters such as urine output and oxygen saturation). The AVPU (A, alert; V, reacting to voice; P, reacting to pain; U, unresponsive) scale used in MEWS corresponds to distinct GCS ranges with overlap between some ranges (Kelly *et al* 2004, Romanelli and Farrell 2020). To derive a one-to-one correspondence between both scales, we adopted the following mapping: Alert = GCS 14–15; Reacting to voice = GCS 10–13; Reacting to pain = GCS 4–9; Unresponsive = GCS 3.

**Table 1.** Modified early warning score.

MEWS	3	2	1	0	1	2	3
Systolic blood pressure (mmHg)	<70	71–80	81–100	101–199		≥200	
Heart rate (bpm)		<40	41–50	51–100	101–110	111–129	≥130
Respiratory rate (bpm)		<9		9–14	15–20	21–29	≥30
Temperature (°C)		<35		35–38.4		≥38.5	
AVPU score^a^				Alert	Reacting to voice	Reacting to pain	Unresponsive

^a^ AVPU: A, alert; V, reacting to voice; P: reacting to pain; U, unresponsive.

When EHR is used as the source of physiological parameter measurements, evaluating MEWS at regular intervals may be challenging since only a subset of measurements might be available at any sampling time point. Two approaches can generally be used to calculate MEWS. In a first approach, scores are calculated at a prescribed regular time interval and missing measurements are imputed. In a second approach, scores are calculated only when a new measurement of one or more parameters is available.

**Figure 1.**
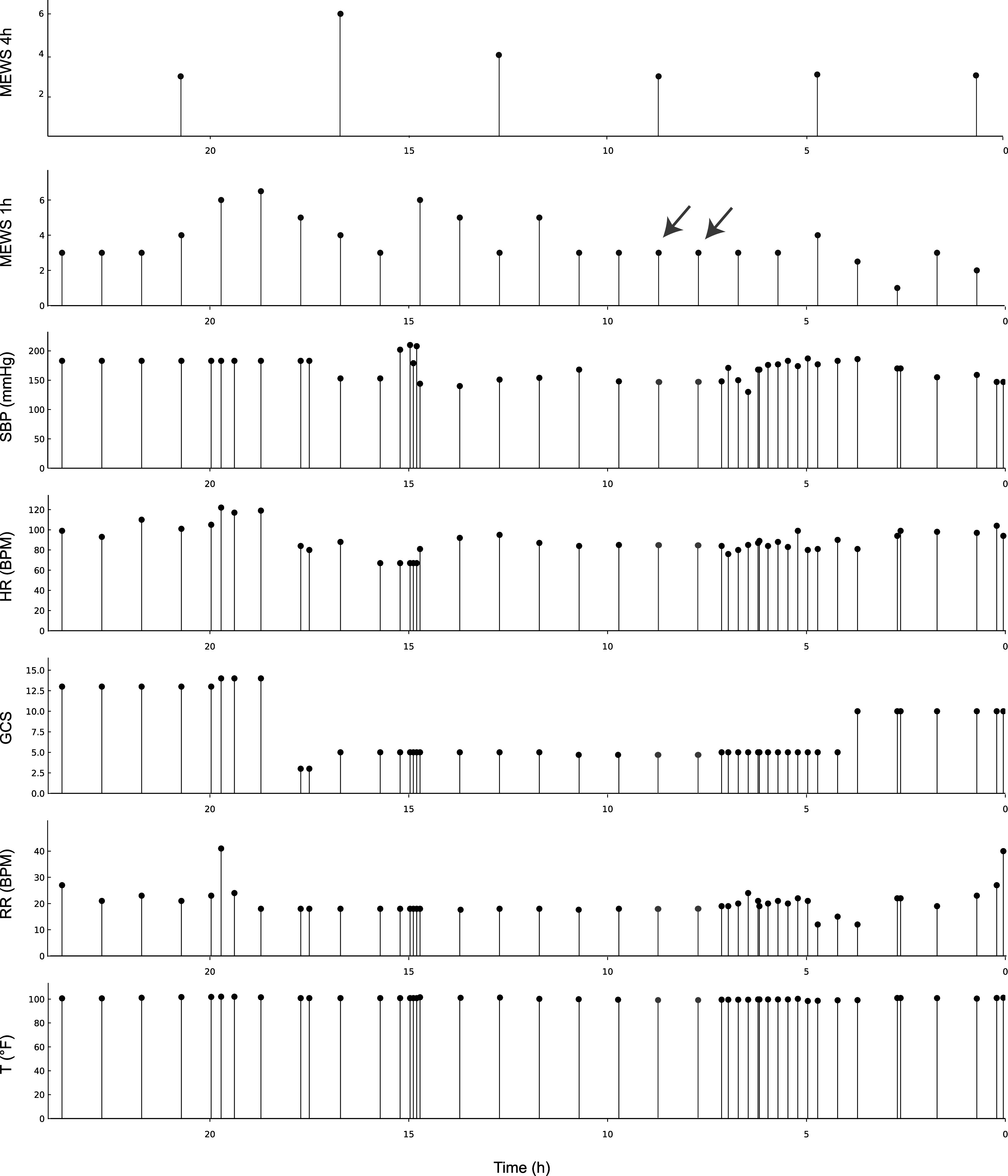
Derivation of a regularly sampled MEWS from physiological parameters. First and second top panels illustrate MEWS values calculated in one patient’s 24 h recording, using T_MEWS_ = 4 h and T_MEWS_ = 1 h, respectively. MEWS was calculated using the median of physiological measurements within T_MEWS_. Missing measurements were imputed to calculate MEWS at 1 h intervals (blue arrows indicate two MEWS values calculated from imputed physiological values, derived by carrying forward the last available measurement, indicated in blue lines).

The details of how MEWS is calculated from irregularly sampled physiological parameters and how missing values are imputed is seldom described in the literature (Subbe *et al* 2001, 2003, Churpek *et al* 2012, Cooksley *et al* 2012, Fullerton *et al* 2012, Drower *et al* 2013, van Rooijen *et al* 2013, Bulut *et al* 2014, Kim *et al* 2015, Mathukia *et al* 2015, Kruisselbrink *et al* 2016, Jayasundera *et al* 2018, Al-Kalaldeh *et al* 2019). A straightforward approach is to calculate a new MEWS value every time a physiological parameter gets refreshed, carrying forward the values of each parameter until a new value is available. This results in an irregularly sampled MEWS, referred to hereafter as MEWS_base_ (this is a common approach of calculating MEWS). Another approach is to calculate MEWS at prescribed regular time intervals, T_MEWS_, using a statistical summary of the available measurements for a physiological parameter within a time interval. We calculated MEWS and evaluated its performance for two different lengths of T_MEWS_ (2 and 12 h) and two different statistics: the median (MEWS_median_) and the worst value of the available physiological measurements within T_MEWS_ (MEWS_worst_). When no new measurements are available within T_MEWS_, MEWS can be calculated using imputed physiological parameter values. Missing data in each physiological parameter were imputed by carrying forward the last recorded value until replaced with a new measurement. Figure 1 illustrates this approach. We implemented these variations of MEWS calculation to test if the proposed validation approaches could reveal whether and how these MEWS implementations would lead to different performances.

MEWS was calculated in the case and control groups according to the rules in table 1. Except for GCS which may not have been recorded for every patient, MEWS was not calculated (and patient was excluded from the analysis) when no measurements were available for a given vital sign in a patient’s data.

### 2.3. Performance evaluation

We describe below two approaches for evaluating clinical prediction indices, using MEWS as a convenient example score to illustrate the concepts and the metrics introduced.

#### 2.3.1. Patient-level evaluation

In many studies evaluating implementations of MEWS, the number of MEWS threshold crossings—given a fixed threshold value—were collectively evaluated with regards to correct prediction of an event (Lee *et al* 2008, Cooksley *et al* 2012, Fullerton *et al* 2012, Bulut *et al* 2014). That is, true predictions (or true positives) are clinical events for which MEWS crossed a threshold, regardless of how many times this occurred. When MEWS crosses a threshold in a patient who did not experience clinical deterioration during his/her hospitalization period it leads to a false positive, regardless of the number of times the threshold was crossed. Clinical deterioration events for which MEWS did not cross a threshold are missed predictions (or false negatives). Finally, true negatives are patients who did not experience clinical deterioration by the end of their hospitalization and in whom MEWS did not cross a threshed (table 2).

**Table 2.** Definition of a confusion matrix in a patient-level evaluation of MEWS.

	Clinical event occurred	No clinical event occurred
One or more alarms triggered	True positive	False positive
No alarm triggered	False negative	True negative

**Figure 2.**
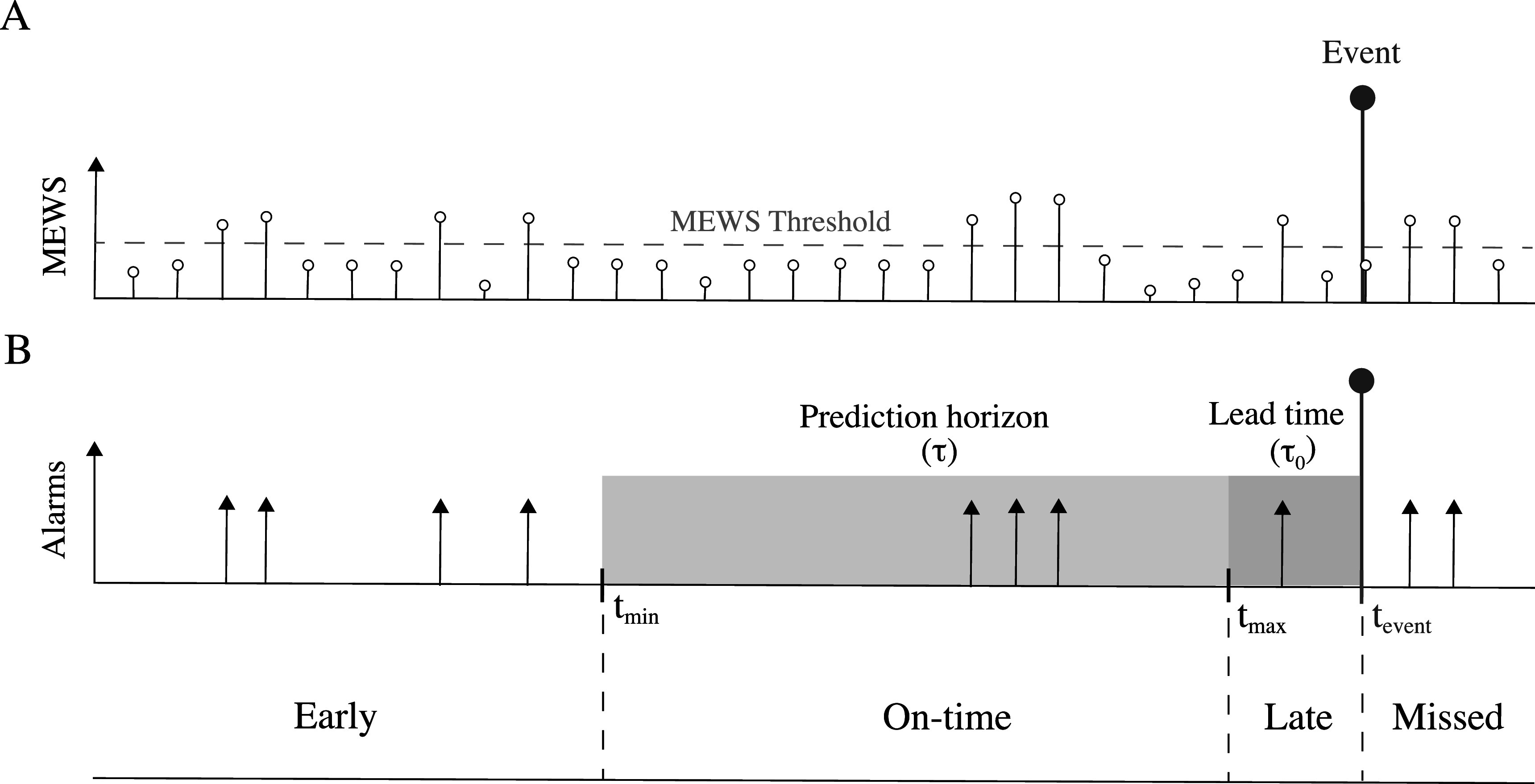
Definition of MEWS alarms, prediction horizon $\tau \,$= [*t*_min_ *t*_max_] and lead time ${\tau }_{0}\,$= [*t*_max_ *t*_*event*_]. An alarm is raised each time MEWS exceeds a threshold (A). *t*_*event*_ is the time of a clinical event, e.g. a cardiac arrest. Alarms are considered early, on-time, late, and missed if they respectively occur before the start of prediction horizon, within the prediction horizon, within the lead time, and after the onset of the clinical event. Early, late and missed alarms are false alarms (B).

In a system where MEWS threshold crossings translate into MEWS alarms, the above definitions become limited as they do not quantify the timeliness and burden of these alarms. We hereafter refer to the above evaluation scheme as *patient-level* evaluation and propose an *event-level* evaluation by considering the timeliness and the temporal distribution of MEWS alarms to quantify the burden and practicality of notifications. MEWS alarms can be defined as events triggered and cleared following a set of rules. Here, and to simplify subsequent analysis, we define a MEWS alarm an instantaneous notification occurring when MEWS exceeds a given threshold value (figure 2(A)).

#### 2.3.2. Prediction horizon and lead time

The definition of a temporal window preceding the time of an event allows performance to be evaluated in respect to an actionable timeframe. MEWS alarms have different clinical implications when they occur ‘too early’ or ‘too late’. We therefore define two temporal windows, the *prediction horizon* and the *lead time*, to quantify the timeliness of alarms. A prediction horizon is a time window preceding the time of event (i.e. [*t*_min_ *t*_max_] where *t*_min_ < *t*_max_ < *t*_*event*_), within which alarms are deemed actionable, that is providing sufficient time for caregivers to intervene (figure 2(B)). For code blue events, the length of a prediction horizon $\tau ={t}_{{\mathrm{\max }}}-{t}_{{\mathrm{\min }}}$ could be defined according to typical ICU nurse’s shift (e.g. 12 h).

*t*_min_ is the earliest time (with respect to *t*_*event*_) an alarm can contribute to true predictions. By varying *t*_min_ and setting *t*_max_ = *t*_min_ + $\tau ,$ we can create a realistic and clinically meaningful characterization of the ability of MEWS to predict code blue events in the ICU. Applying such definitions of *t*_min_ and *t*_max_ is a general approach and allows performance characterization at a lead time before the onset of event, defined by *t*_max_, where the lead time is the interval of time [*t*_max_ *t*_*event*_]. The duration of the lead time ${\tau }_{0}$ serves as the minimum time window required for an intervention to be effective. Any alarm occurring within the lead time is a late alarm. It is considered either a ‘short-notice’ or a redundant alert if it is preceded by one or more alarms triggered within the prediction horizon. Alarms generated after event onset (*t*_*event*_) are deemed missed alarms. Early, late, and missed alarms are all false alarms.

#### 2.3.3. Event-level evaluation

Given the definitions of prediction horizon and lead time, we derive a series of time-dependent metrics to evaluate the performance of MEWS and the burden of alarms. Table 3 summarizes these metrics and their definitions.

**Table 3.** Summary of proposed metrics to evaluate a predictive alarm system.

Metric	Definition	Rationale	Formula
Time-dependent sensitivity ${S}^{\tau ,{\tau }_{0}}$	Proportion of events preceded by at least one alarm within a prediction horizon	This metric quantifies the proportions of predicted events given a fixed time window within which MEWS scores above a threshold are considered true alarms, and outside of which they are considered false alarms	${S}^{\tau ,{\tau }_{0}}=\tfrac{{N}_{predicted\,events}}{{N}_{events}}$
			${N}_{predicted\,events}:\mathrm{Number}\,\mathrm{of}\,\mathrm{predicted}\,\mathrm{events}$
			${N}_{events}:\mathrm{Total}\,\mathrm{Number}\,\mathrm{of}\,\mathrm{events}$
False positive ratio (FPR)	Proportion of the expected number of control patients in whom alarms are triggered within a window of a length equal to the prediction horizon	This metric estimates the proportion of control patients who are likely to trigger a false alarm. This estimate is based on randomized occurrences of a time window within which MEWS scores above a threshold are deemed false alarms	${\hat{\mu }}_{FPR}=\tfrac{\displaystyle {\sum }_{i=1}^{N}\displaystyle {\sum }_{j=1}^{M}{T}_{ij}}{N.M}$
			*N*: Number of control patients
			*M*: Number of trials to estimate FPR (e.g.* M* = 1000)
			*T*_*ij*_: *j*th randomly selected time window from the control data of the *i*th control patient
			${T}_{ij}=\left\{\begin{array}{l}1,\,\,\,an\,alarm\,is\,triggered\,within\,{T}_{ij}\\ 0,\,\,otherwise\end{array}\right.$
Alarm rate (*r*)	Number of alarms per unit time	This is a measure of the burden of alarms, including true and false alarms	$r=\tfrac{{N}_{alarms\,}}{{T}_{recording}}$
			${N}_{alarms}:{\mathrm{Number}}\,{\mathrm{of}}\,{\mathrm{alarms}}$ in a patient recording data
			${T}_{recording}:{\mathrm{Recording}}\,{\mathrm{data}}\,{\mathrm{duration}}$
Alarm proportion $(\rho )$	Proportion of score samples triggering an alarm	This is measure of the alarm burden. It quantifies the relative number of score samples triggering an alarm	$\rho =\tfrac{{N}_{MEWS\,> \,k}}{{N}_{MEWS\,\geqslant \,0}}$
			${N}_{MEWS\,> \,k}:{\mathrm{Number}}\,{\mathrm{of}}\,{\mathrm{MEWS}}\,{\mathrm{samples}}\,{\mathrm{above}}\,{\mathrm{thrshold}}\,k$
			${N}_{MEWS\,\geqslant \,0}:$ Number of MEWS samples
False alarm rate (*r* ^0^)	Number of false alarms per unit time	Measures the burden of false alarms by estimating their (hourly) frequency. A high rate may lead to alarm fatigue	${r}^{0}\left(case\right)=\tfrac{{N}_{falseAlarms}}{T}$
			${N}_{falseAlarms}:{\mathrm{Number}}\,{\mathrm{of}}\,{\mathrm{false}}\,{\mathrm{alarms}}$
			$T=\left\{\begin{array}{l}{T}_{recording}-\tau :\,for\,case\,patients\\ {T}_{recording}:\,for\,control\,patients\end{array}\right.$
False alarm proportion $({\rho }^{0})$	Proportion of score samples triggering a false alarm	Measures the burden of false alarms by estimating their (relative) quantity. A high proportion may lead to alarm fatigue	${\rho }^{0}=\,\tfrac{{N}_{falseAlarms\,}}{{N}_{MEWS\,\geqslant \,0}}$
Work-up to detection ratio (*WDR*)	Ratio of the numbers of true and false predictions to the number of true predictions events	Measures the number of patients who will receive a workup before one additional adverse outcome can be prevented	$WDR=\tfrac{{N}_{case}\,+\,{\hat{\mu }}_{FP}}{{N}_{case}}$
			*N*_*case:*_ Number of case patients in whom at least one alarm was triggered within a prediction horizon
			${\hat{\mu }}_{FP}:{\mathrm{Estimated}}\,{\mathrm{number}}\,{\mathrm{of}}\,{\mathrm{false}}\,{\mathrm{positives}}$
Time profile of alarm proportion	Normalized summary histogram of alarms with respect to number of alarms	An estimate of the probability of an alarm triggered early, on-time, late, or missing an event	N/A
Time profile of alarms per event	Normalized summary histogram of alarms with respect to number of events	Time profile of alarm proportion with respect to events	N/A

##### 2.3.3.1. Time-dependent sensitivity

Alarms triggered within the prediction horizon are defined as true alarms and alarms triggered outside of the prediction horizon (before or after) as false alarms. To quantify the sensitivity of MEWS given these definitions, we consider an event to be successfully predicted if it was preceded by at least one true alarm (Notice that a distinction is made between a true alarm and a true positive. A ‘positive’ refers a code blue event just like it was defined in the patient-level evaluation.) This sensitivity depends on the choice of $\tau \,{\mathrm{and}}\,{\tau }_{0}$ and its formally defined as the proportion of clinical events for which at least one alarm was triggered within the prediction horizon:

\begin{eqnarray*}{S}^{\tau ,{\tau }_{0}}=\displaystyle \frac{{N}_{predicted\,events}}{{N}_{events}},\end{eqnarray*}

where ${N}_{predicted\,events}$ is the number of predicted clinical events and ${N}_{events}$ is the total number of clinical events.

##### 2.3.3.2. Time-dependent specificity

Event-level specificity metrics can be derived from case and control records. We first propose to estimate the false positive ratio (FPR) in the control group, defined as the ratio of the expected number of control patients in whom alarms are triggered within a window of a length equal to the prediction horizon, to the total number of control patients *N* (Bai *et al* 2015). Let ${\hat{\mu }}_{FPR}$ be the expected the estimated value of FPR. ${\hat{\mu }}_{FPR}$ can be calculated using a bootstrapping approach. For each control patient *i* (1 $\leqslant $ *i* $\leqslant $ N), we randomly sample a window of length $\tau $ over its whole monitoring time. This is repeated a sufficiently large number of time *M* (e.g. *M* = 1000). The windows are then temporally sorted and indexed using their temporal order (the *j*th window is preceded in time by the (*j*−1*)*th window). We then calculate the number of windows in which one or more alarms occurred (triggered windows) to estimate the expected value of whether the patient counts as a false positive.

Let *T*_*ij*_ = 1 (1 $\leqslant $ *i* $\leqslant $ *N*; 1 $\leqslant $ *j* $\leqslant $ *M*) if the *j*th window selected for the *i*th control patient gets triggered, and *T*_*ij*_ = 0 otherwise. The expected number of control patients ‘triggering’ a *j*th window is:

\begin{eqnarray*}{\hat{\mu }}_{FP}^{j}=\displaystyle \sum _{i=1}^{N}{T}_{ij}\end{eqnarray*}

and its standard deviation is:

\begin{eqnarray*}{\hat{\sigma }}_{FP}^{j}=\sqrt{\displaystyle \frac{\displaystyle {\sum }_{i=1}^{N}{\left({T}_{ij}-{\hat{\mu }}_{FP}^{j}\right)}^{2}}{N-1}}.\end{eqnarray*}

The estimated value ${\hat{\mu }}_{FPR}$ and its standard deviation can then be calculated as:

\begin{eqnarray*}{\hat{\mu }}_{FPR}=\displaystyle \frac{\displaystyle {\sum }_{j=1}^{M}{\hat{\mu }}_{FP}^{j}}{M}=\displaystyle \frac{\displaystyle {\sum }_{i=1}^{N}\displaystyle {\sum }_{j=1}^{M}{T}_{ij}}{N.M},\end{eqnarray*}

\begin{eqnarray*}{\hat{\sigma }}_{FPR}=\sqrt{\displaystyle \frac{\displaystyle {\sum }_{j=1}^{M}{\left({\hat{\sigma }}_{FP}^{j}\right)}^{2}}{M}}.\end{eqnarray*}

Other metrics (accuracy, positive and negative predictive values and F1 score) can be calculated using the same bootstrapping approach (see supplementary material S1 (available online at stacks.iop.org/PMEA/42/055005/mmedia)). To quantify the specificity of MEWS in the case groups, we propose to calculate the proportion and rate of alarms and false alarms which also quantify the alarm burden.

##### 2.3.3.3. Alarm burden

The number of alarms generated during an ICU stay in case patients can be summarized by visualizing the distribution of the alarms that occur ‘early’, ‘late’, and ‘on-time’, and alarms that were ‘missed’, with respect to the prediction horizon and time of event. To quantify these distributions, alarms from all case patients are summed up within each of the four attributed windows to create a *time profile or alarms*. This profile may be interpreted as the probability distribution of generating an alarm early, on-time, late, or missing an event. The number of alarms can further be normalized by the total number of alarms or by the total number of events resulting in a *time profile of alarms per event* and a *time profile of alarm proportions*, respectively (Scully and Daluwatte 2017).

Additionally, we calculate the rate of alarms and false alarms (*r* and *r* ^0^, resp.), and the proportion of alarms and false alarms ($\rho $ and ${\rho }^{0},$ resp.) to measure the burden of alarms and false alarms. *r* and *r* ^0^ measures the number of alarms and false alarms, respectively, per unit time per patient.

###### 2.3.3.3.1. Rate and proportion of alarms

For a given patient in the case or control group*, r* and $\rho $ are given by:

\begin{eqnarray*}r=\displaystyle \frac{{N}_{alarms\,}}{{T}_{recording}\,},\end{eqnarray*}

\begin{eqnarray*}\rho =\displaystyle \frac{{N}_{alarms\,}}{{N}_{MEWS}\,}=\displaystyle \frac{{N}_{MEWS\,> \,{k}}}{{N}_{MEWS\,\geqslant \,0}},\,0\leqslant {k}\leqslant 13,\end{eqnarray*}

where *N*_*alarms*_ is the number of MEWS above a threshold *k* and *N*_*MEWS*_ is the number of MEWS samples (equivalently, the number of MEWS above threshold *k* = 0) in the patient recording.

###### 2.3.3.3.2. Rate and proportion of false alarms

A false alarm is an alarm that occurs outside of the prediction horizon by the previous definitions. Hence, the false alarm rate for a given patient in the case group is given by:

\begin{eqnarray*}{r}^{0}\left(case\right)=\displaystyle \frac{{N}_{falseAlarmsCase}}{{T}_{recordingCase}\,-\,\tau }.\end{eqnarray*}

With ${N}_{falseAlarmsCase}$ is the total number of false alarms, and ${T}_{recordingCase}$ the duration of the recording. Here, the duration of the prediction horizon is subtracted from the total duration of recording since a false alarm cannot occur within a prediction horizon by definition.

To calculate *r* ^0^ for a control patient, we simply derive the average rate of alarms triggered for a control patient:

\begin{eqnarray*}{r}^{0}\left(control\right)=\displaystyle \frac{{N}_{falseAlarmsControl}}{{T}_{recordingControl}},\end{eqnarray*}

where ${N}_{falseAlarmsControl}$ is the total number of false alarms and ${T}_{recordingControl}$ the duration of the recording in a given control patient.

By substituting ${N}_{falseAlarmsCase}$ and ${N}_{falseAlarmsControl}$ in the numerator of equation (2.7) we get the proportion of false alarms ${\rho }^{0}(case)$ and ${\rho }^{0}(control)$ in the case and control group, respectively.

##### 2.3.3.4. Work-up to detection ratio (*WDR*)

Furthermore, we introduce the *WDR* ratio to evaluate the effectiveness of MEWS. *WDR* is defined as the ratio of the number of case patients *N*_*case*_ in whom at least one alarm was triggered within a prediction horizon (equivalently the number of predicted events) and the number of control patients in whom a random window of length $\tau $ is triggered (i.e. the estimated number of false positives, ${\hat{\mu }}_{FP}$), to the number of predicted events:

\begin{eqnarray*}WDR=\displaystyle \frac{{N}_{case}+{\hat{\mu }}_{FP}}{{N}_{case}}.\end{eqnarray*}

Conceptually, *WDR* is similar to the number needed to alert, defined as the number of alerts that need to be reviewed to detect one potential adverse event (Moore *et al* 2009). WDR measures the number of required workups to prevent one additional adverse outcome (equivalently, the number of case patients to be treated for one of them to benefit from the treatment compared with a control patient).

Using (2.4), and (2.5), *WDR* and its standard deviation can be estimated as:

\begin{eqnarray*}{\hat{\mu }}_{WDR}=1+\displaystyle \frac{\displaystyle {\sum }_{i=1}^{N}{\hat{\mu }}_{i}}{{N}_{case}},\end{eqnarray*}

\begin{eqnarray*}{\hat{\sigma }}_{WDR}=\displaystyle \frac{\sqrt{\displaystyle {\sum }_{i=1}^{N}{{\hat{\sigma }}_{i}}^{2}}}{{N}_{case}}.\end{eqnarray*}

## 3. Results

27 of 283 case patients (9.5%) and 6 of 3127 control patients (0.2%) were excluded from the analysis because MEWS could not be calculated (due to missing of one or more vital signs) and/or because the duration of the recording was less than the prediction horizon $\tau =12\,{\mathrm{h}}$ for the sensitivity to be evaluated in case patients and FPR to be estimated in control patients. This led to 7% prevalence of code blue events.

MEWS_base_ was evaluated using the traditional metrics (AUC, accuracy, sensitivity, specificity, and precision). We then evaluated the impact of calculating MEWS from summary statistics of physiological measurements in comparison with MEWS_base_. Finally, we evaluate MEWS using the proposed metrics. The prediction horizon $\tau $ was set to 12 h—typical length of nursing shift—and the lead time ${\tau }_{0}$ was varied between 0 and 6 h at 10 min increments. Case patients for whom the length of ICU stay was less than the sum of lead time and seizure prediction horizon (i.e. length of stay < $12\,{\mathrm{h}}+{\tau }_{0}$) were excluded from the relevant analysis since true predictions could not be evaluated. The number of excluded patients increased linearly from 0 (for ${\tau }_{0}\,\,$= 0) to 38 (for ${\tau }_{0}\,\,$= 6 h). MEWS was evaluated for threshold values *k* varying between 0 and 13. Table 4 describes the frequency of vital signs and GCS measurements and the proportion of imputed values in the dataset.

**Table 4.** Average sampling rate (measurements per hour) of physiological parameters and proportion of missing values in the case and control groups.

		GCS	Temp	SBP	RR	HR
Case	Average sampling rate (1/h)	0.3	1	1.2	2	2.2
	Proportion of imputed values	0.9	0.5	0.5	0.1	0
Control	Average sampling rate (1/h)	0.1	0.4	0.5	0.7	0.8
	Proportion of imputed values	0.9	0.6	0.4	0.1	0.1

### 3.1. Patient-level and event-level evaluation of MEWS_base_

Table 5 shows the performance of MEWS_base_ evaluated using classic metrics applied to the patient-level and event-level approaches (see sections 2.3.1 and 2.3.3, resp.). MEWS threshold *k* was set to 4, a commonly used threshold value (Subbe *et al* 2001, Burch *et al* 2008).

**Table 5.** Performance of MEWS using patient-level and event-level evaluations. MEWS threshold was set to *k* = 4.

Evaluation	TPR	FPR (std)	NPV (std)	PPV (std)	ACC (std)	F1 (std)	WDR (std)
Patient-level	0.97	0.77	0.99	0.09	0.29	0.17	10.7
Event-level ($\tau $ = 12 h, ${\tau }_{0}$ = 0, *M* = 1000)	0.87	0.30 (0.14)	0.98 (0.00)	0.23 (0.12)	0.71 (0.13)	0.35 (0.13)	5.3 (1.94)

Note. TPR: True Positive Rate (Sensitivity); FPR: False Positive Rate; NPV: Negative Predictive Value; PPV: Positive Predictive Value; ACC: Accuracy. WDR: Workup-to-detection ratio.

**Figure 3.**
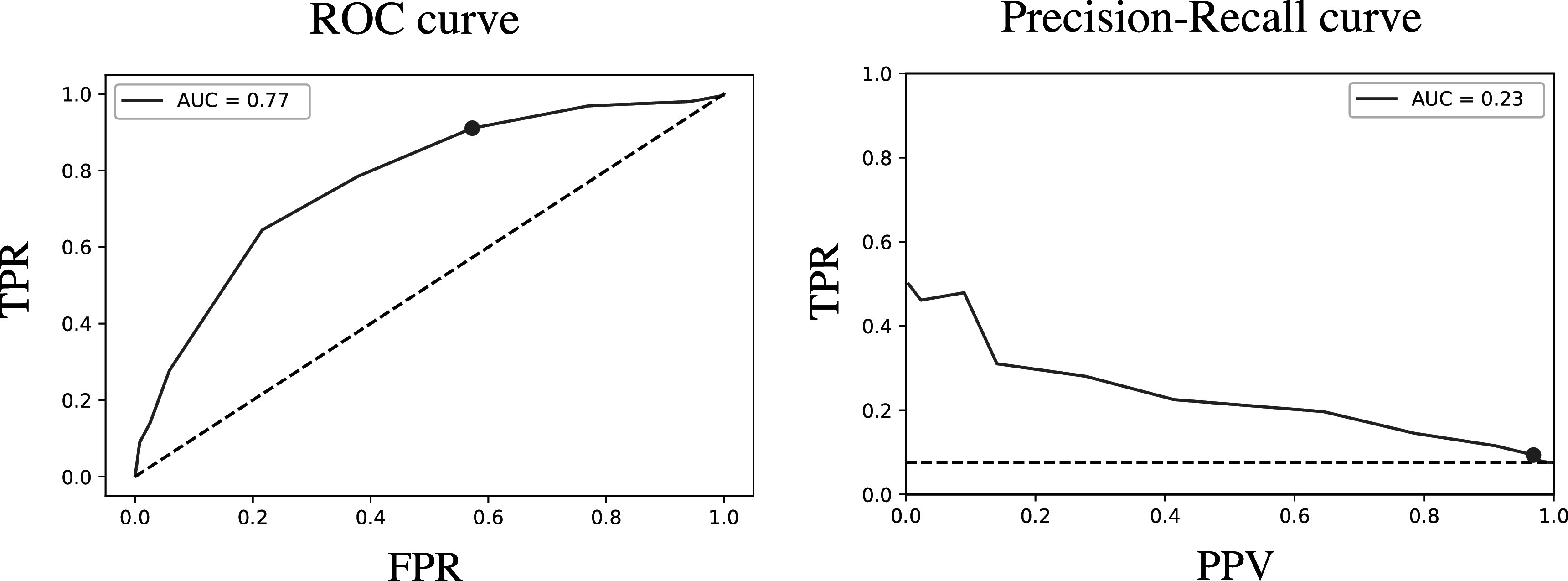
ROC curve (left) and precision-recall (right) curve of MEWS performance evaluated for 14 discrete threshold levels (*k* = 0–13) using the patient-level evaluation approach. The area under the curve (AUC) is a measure of MEWS performance. Dashed lines represent chance level. Red dots indicate the performance of MEWS at threshold *k* = 4.

Using a patient-level evaluation, the high sensitivity of MEWS (TPR = 0.97) was significantly compromised by low precision (0.09) and low specificity (FPR = 0.77). Figure 3 depicts the performance of MEWS for each of the 14 possible threshold values using a Receiver operating characteristic (ROC) curve and the precision-recall curve (PRC).

Using an event-level approach with a prediction horizon $\tau \,$= 12 h and a lead time ${\tau }_{0}\,$= 0 h in the case group, and 1000 random windows in the control group, a substantial improvement in the precision (∼155%), the FPR (∼60%) and in the WDR (∼50%) could be achieved in the expense of a relatively marginal decrease in sensitivity (∼10%).

**Figure 4.**
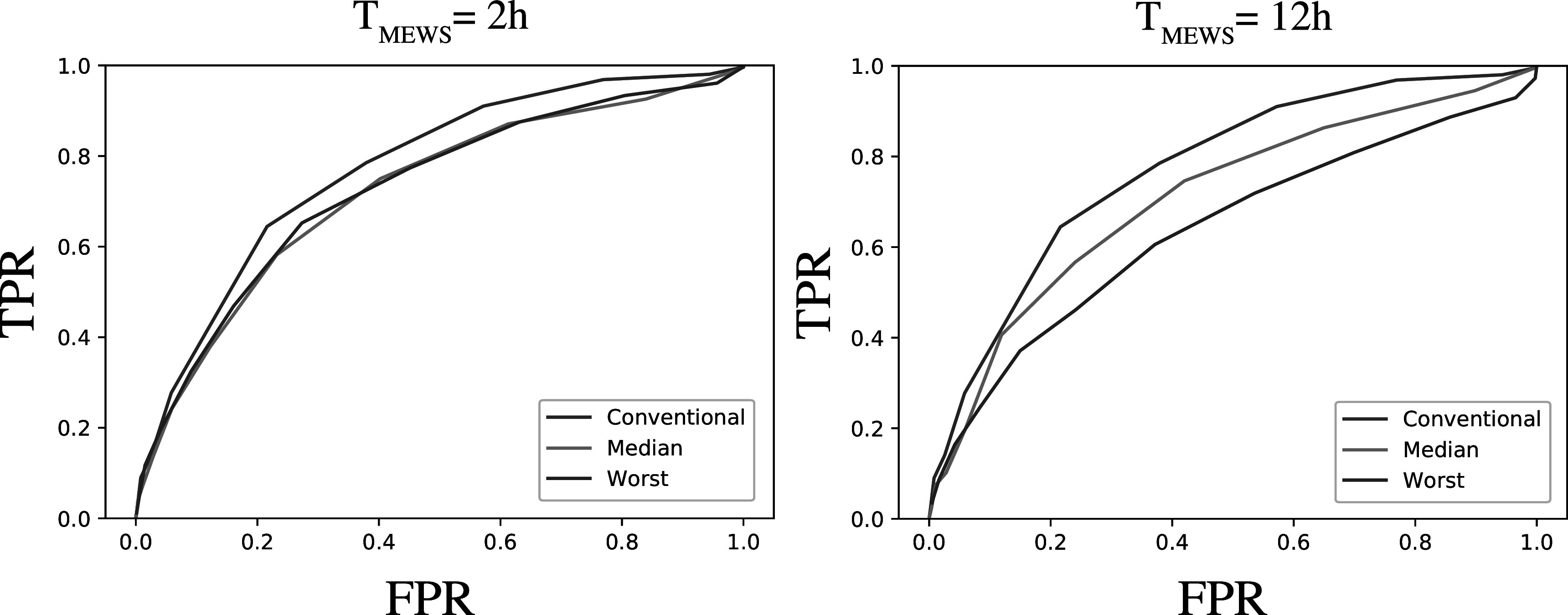
Performance of MEWS calculated at irregular time points (MEWS_base_) and at regular time intervals T_MEWS_ = 2 and 12 h using the median (MEWS_median_) and the worst value of physiological measurements within T_MEWS_ (MEWS_worst_). MEWS threshold was set to *k* = 4.

The performance of MEWS calculated at regular time intervals was lower than that of the MEWS_base_ (measured by AUC) regardless of the length of T_MEWS_ and the statistic used (figure 4). MEWS_median_ was less affected by the length of T_MEWS_ than MEWS_worst_, which performance degraded using a T_MEWS_ of 12 h compared to a T_MEWS_ of 2 h.

### 3.2. Time-dependent sensitivity

**Figure 5.**
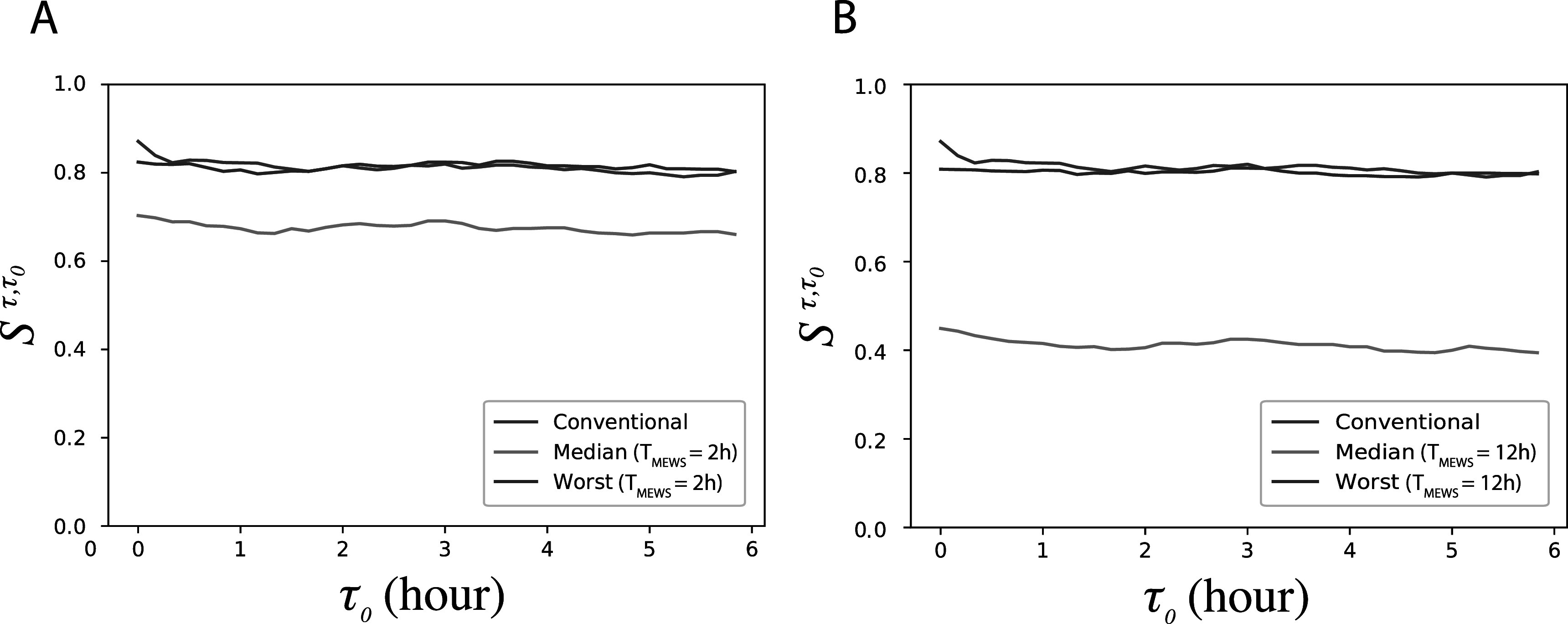
Sensitivity ${S}^{\tau ,{\tau }_{0}}$ of MEWS evaluated for a prediction horizon $\tau \,$= 12 h and lead time ${\tau }_{0}$ varied between 0 and 6 h at 10 min increments. MEWS was calculated at irregular time points (conventional or MEWS_base_) and at regular time intervals T_MEWS_ = 2 h (A) and 12 h (B) using the median (MEWS_median_) and the worst value of physiological measurements within T_MEWS_ (MEWS_worst_). MEWS threshold was set to *k* = 4.

Setting MEWS threshold to *k* = 4 and the prediction horizon to $\tau \,$= 12 h, there was no significant change in the sensitivity when the lead time was varied between 0 and 6 h (see figure 5). The sensitivity of MEWS_median_ was significantly lower than that of MEWS_worst_ and MEWS_base_ (t-test, *p* < 0.05) when T_MEWS_ was set to 2 or 12 h, and for different lead time values (see section 2.3.3.1). MEWS_base_ and MEWS_worst_ showed comparable sensitivities for either value of T_MEWS_.

### 3.3. Alarm burden

#### 3.3.1. Alarm time profile

**Figure 6.**
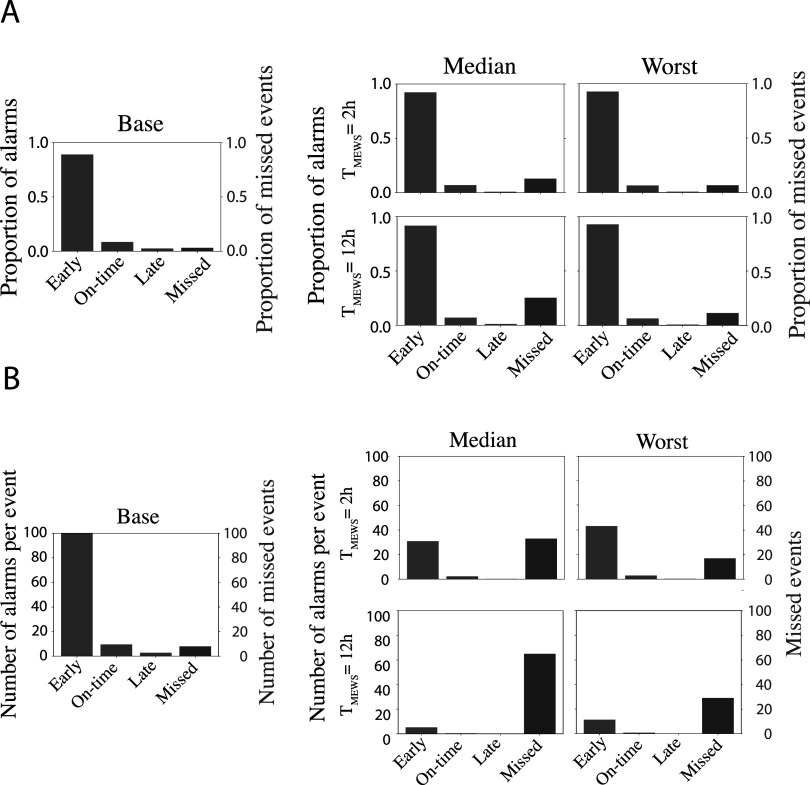
Time profile of alarms and alarms per event calculated in case patients for 3 different calculation methods of MEWS and 2 values of T_MEWS_. (A) Proportion of early, on-time, and late alarms and missed events. (B) Number of early, on-time and late alarms per event and number of missed events. MEWS threshold was set to *k* = 4. Prediction horizon was set to $\tau \,$= 12 h and lead time was set to ${\tau }_{0}=$ 1 h.

Figure 6 illustrates the distribution of the proportion of alarms and alarms per event (see section 2.3.3.3) across four time periods. Regardless of the length of T_MEWS_ and how MEWS is calculated, most alarms were triggered early, before the start of the pre-defined prediction horizon. The proportion of missed events is relatively lower with MEWS_base_ than with MEWS_Median_ or MEWS_worst_, for both T_MEWS_ values. Longer T_MEWS_ results into higher proportion of missed events and a lower rate of early and on-time alarms per event for both MEWS_Median_ and MEWS_worst_.

#### 3.3.2. False alarm rate and proportion of false alarms (see section 2.3.3.3.2)

**Figure 7.**
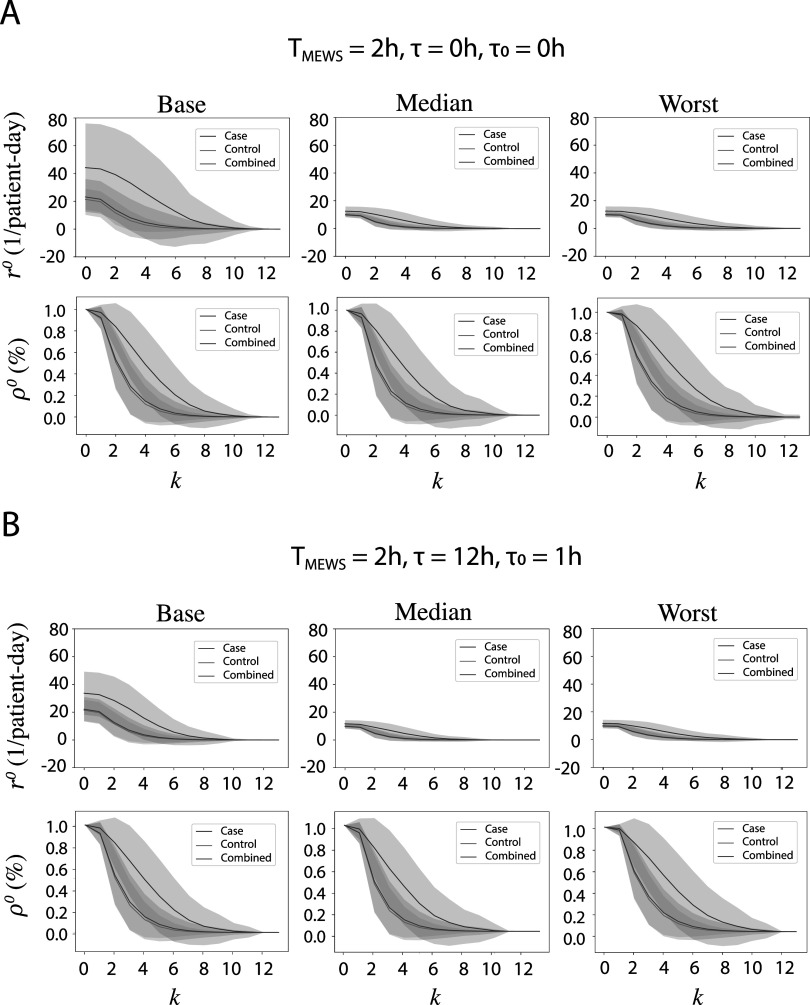
False alarm rate and proportion of false alarms in the case and control groups, and in the combined population for different MEWS thresholds *k*, T_MEWS_ = 2 h, and two different value sets of the prediction horizon and the lead time: (A) $\tau \,$ = 0 h, ${\tau }_{0}\,$ = 0 h and (B) $\tau $ = 12 h, ${\tau }_{0}\,$ = 1 h. Shadows indicate one standard deviation of the mean.

We set the values of prediction horizon $\tau $ and lead time ${\tau }_{0}$ to 0 in order to evaluate the effect of calculating MEWS with different methods on the burden of false alarms. The average false alarm rate and average proportion of false alarms across patients were generally highest for MEWS_base_ and decayed exponentially with increasing values of *k* (figure 7(A)). At *k* = 4 and T_MEWS_ = 2 h, the average combined (case and control) proportion of false alarm ${\rho }^{o}$ and the average combined false alarm rate *r* ^0^ were significantly higher (t-tests, *p* <0.05) for MEWS_base_ (19% and 0.19/h, resp.) compared to MEWS_median_ (12% and 0.05/h resp.). MEWS_worst_ resulted in significantly lower *r* ^0^ (0.08/h) but not ${\rho }^{o}$ (18%) compared with MEWS_base_.

At $\tau \,$ = 12 h and ${\tau }_{0}$ = 1 h, both *r* ^0^ and ${\rho }^{o}$ decayed similarly as for $\tau \,$ = ${\tau }_{0}$ = 0 h (figure 7(B)). For *k* $\geqslant $ 4 any improvement in *r* ^0^ and ${\rho }^{o}\,$ is arguably marginal. Highest values of *r* ^0^ and ${\rho }^{o}\,$ were observed for MEWS_base_ overall.

#### 3.3.3. Rate and proportion of alarms (see section 2.3.3.3.1)

At $\tau \,$ = 0 and ${\tau }_{0}$ = 0, the average alarm rate decayed exponentially with MEWS threshold as was the case of false alarm rate. MEWS_base_ generates the highest alarm rate at any MEWS threshold value compared with MEWS_median_, and MEWS_worst_, which both led to comparable alarm rates for all values of *k* and T_MEWS_ values and larger alarm rates for longer T_MEWS_ values. Average alarm rates were expectedly higher in the case group than in the control group.

The proportion of MEWS values above a given threshold (alarm proportion) was relatively comparable for all MEWS calculation methods and T_MEWS_ values. Compared with conventional MEWS, the alarm rate decreased by ∼70% (from 13.68/patient-day to 4.08/patient-day) in the case group for T_MEWS_ = 2 h, and by 95% for T_MEWS_ = 12 h (from 13.68/patient-day to 0.37/patient-day). This translates into a reduction in the hourly alarm rate from ∼1 alarm every 1.7 h to ∼1 alarm every 5.8 h, and to 1 alarm every 33 h, for T_MEWS_ = 2 h and 12 h, respectively. Table 6 provides a summary of alarm proportions and alarm rates for *k* = 4.

**Table 6.** Number of MEWS samples, alarm rate (*r*) and alarm proportion ($\rho $), in the case and control group. For different MEWS calculation methods. Average numbers shown. MEWS threshold was set to *k* = 4.

	MEWS_Base_				MEWS_Median_		MEWS_Worst_	
	Case	Control			Case	Control	Case	Control
*Number of MEWS samples (*1/*patient-day)*	51.98	21.50	T_MEWS_ (h)	2	14.17	9.61	14.17	9.61
				12	5.70	2.10	5.70	2.10
*r (*1/*patient-day)*	13.68	4.08	T_MEWS_ (h)	2	4.08	1.2	5.52	2.16
				12	0.72	0.24	1.44	0.72
$\rho $ (%)	0.46	0.19	T_MEWS_ (h)	2	0.37	0.13	0.52	0.22
				12	0.33	0.11	0.72	0.35

## 4. Discussion

The clinical importance of predictive alarm systems as risk management tools to help prevent in-hospital patient deterioration has been discussed in numerous systematic reviews (Robert *et al* 1999, McNeill and Bryden 2013, Vincent *et al* 2018, Kramer *et al* 2019, Gerry *et al* 2020). Many of these reviews highlighted the methodological weaknesses and questioned the true predictive power of these systems. Methodological and statistical evaluations that consider among other aspects the temporal dynamic of the measure profile (in the case of continuous monitoring), the timeliness of alarms, and frequency of false alarms can aid in this evaluation. In this study, we investigated the performance a widely adapted EWS, MEWS, using a new set of metrics that capture two critical aspects of any predictive alarm algorithm, namely the timeliness and the burden of alarms. We adjusted the definition of sensitivity to take into account two clinically important time intervals, the prediction horizon and the lead time. These parameters can help provide performance information that may be more informative on clinical utility, since an alarm is arguably useful if it’s *actionable* (occurs within a nursing shift) and *intervenable* (provides a minimum time for an intervention to be effective). This proposed definition of sensitivity informs whether a continuous predictive alarm algorithm can practically identify patients at risk of deterioration and alert caregivers early enough to intervene. The characteristic curve of the sensitivity versus lead time can guide the choice of operational lead time values.

This study aims at proposing a general framework of methods and metrics for evaluating the performance of early warning scoring and predictive algorithms. The intended framework is sought to engage researchers in testing the predictive power of a proposed algorithm under different conditions and alarm protocols to emulate the realm of real-word clinical setting. Parameters such as the prediction horizon and the lead time can be applied universally to test the performance of any predictive alarm algorithm operating on continuous data. Moreover, a variety of alarm triggering strategies can be envisaged and tested to assess if they influence the clinical burden.

### 4.1. Limitations

This study has limitations. While patients were selected consecutively to avoid a selection bias, some case patients had significantly shorter recordings than others. Some metrics (e.g. proportion of early alarms) may have been biased for these patients leading to lower values than what could be observed if recordings had the same length. Estimating specificity-metrics (proportion of false alarms, false alarm rate) over recordings of equal length rather would have introduced a patient selection bias. To overcome these limitations, an alternative approach would be to normalize the number of false alarms by the duration of time preceding the prediction horizon (e.g. 10 false alarms triggered within 1 h would contribute the same proportion of early alarms as 100 false alarms triggered within a 10 h timeframe).

Constraining the timeframe in which an alarm is counted a true alarm to a prescribed horizon while relaxing the period of time alarms are labeled as false inherently biases the proportion of false to true alarms. The false alarm rate may not be affected if we assume that the probability of false alarm occurrence is independent of time-to-event. Such an assumption may not be (always) true though. For example, a MEWS system in which the threshold is adjusted as information about the patients’ health status matures with the length of stay may result in fewer (hourly) false alarms generated towards the end of stay than false alarms generated early after the admission using the ‘default’ threshold value.

Performance results depend on alarm definition and protocol. Had we grouped multiple threshold crossings to form one alarm, for example by applying a lockout period after each alarm, within which no alarm would be generated, the rate and proportion of alarms obtained (see table 6) would have likely been reduced since less alarms would have been generated. Such reduction could in fact be significant and lessens the alarm burden. Studies which applied a lockout period demonstrated a decrease of 55% in alerts from a predive model for cardiothoracic ICU patients (King *et al* 2012). A recent study demonstrated that the number of alerts could be reduced by 90% when a 15 min lockout period was applied to a prediction algorithm for ICU hypotension events (Yoon *et al* 2020).

EHR data are sparse with irregular time interval, it may be challenging to design rules to group alarms that result in appreciable difference. However, if MEWS was generated from continuous vital signs (i.e. from bedside monitors), the number of MEWS alarms generated would have differed significantly in both alarm approaches and would have led to significant difference in sensitivity and specificity results.

Finally, data imputation through forward carrying of the last available physiological measurement were not constrained to expire after an amount of time in this study. It is worth noting that this practice is safe for imputing missing physiological parameter values that are slow changing in nature, such as temperature, or GCS in the case of MEWS, but may not reflect the true underlying physiological state in the case of other parameters. Limiting the time an imputed value may be carried forward can limit the effect of misrepresenting the physiological state but may result in missing samples (in the case of regular sampling) if no measurements are available after expiration of the imputed physiological parameter value.

A critical step in the development of a predictive algorithm is to statistically validate its performance. That is to demonstrate it has an above-chance predictive power. A key question is whether an adverse event can be predicted and whether an algorithm, which is presumed to perform better than chance, does not have predictive power but simply has not yet been tested against appropriate null hypotheses (Andrzejak *et al* 2009). Different statistical approaches have been proposed to answer this question. These include analytical methods (e.g. comparison with naïve predictors) and Monte Carlo based methods (e.g. alarm surrogates) (Schelter *et al* 2006, Snyder *et al* 2008, Feldwisch-Drentrup *et al* 2011).

### 4.2. On the performance of MEWS

The proposed metrics were applied to measure and evaluate the performance of different implementations of MEWS to predict code blue events. Here, we discuss new insights as provided by the proposed performance metrics relating to the potential clinical performance of MEWS that were missing in prior literature (Fairclough *et al* 2009, Heitz *et al* 2010, Tirotta *et al* 2017, Akgun *et al* 2018, Xie *et al* 2018, Jiang *et al* 2019, Ahn *et al* 2020, Aygun *et al* 2020, Balshi *et al* 2020, Gerry *et al* 2020, Kumar *et al* 2020).

Although we found that the sensitivity did not significantly change with lead time values up to 6 h (see section 3.2), it remains to be investigated if higher sampling rates of physiological measurements (e.g. continuous vital sign recordings from patient monitors) can uncover trending changes in MEWS values that may lead to higher sensitivities at short lead times. Additionally, measurements were sampled on average 2.5 times more frequently in patients of the case group than of the control group to presumably capture sudden physiological changes that are more likely to occur in the sicker patient. Higher sampling rates in the control group may have led to different distributions of MEWS alarms and therefore influenced the calculated performance results. The effect of the sampling rate on the calculated metrics may be less prominent for MEWS_median_ than for MEWS_worst_ and conventional MEWS since MEWS_median_ is the least affected by a change in data fluctuation that may be caused by increasing the number of samples within T_MEWS_.

Despite a high sensitivity (97%) observed using the widely adopted patient-level evaluation approach, the false positive rate and the precision of the conventional MEWS were remarkably poor at a threshold value of 4 (77% and 9%, resp; see section 3.1). One of ten patients who triggered an alarm had a code blue outcome (WDR ∼ 10), while almost 8 of 10 patients were falsely identified as potentially high-risk patients (FPR = 77%). Judging whether these numbers are acceptable amounts to mainly evaluating the clinical burden associated with a false alert for a particular application. Additionally, the rate of alarms was highest for conventional MEWS. By calculating MEWS over 12 h window, the alarm rate dramatically decreased. For example, with MEWS_median_, one alarm would be triggered every 33 h, instead of every 1.7 h in the case of conventional MEWS. A substantial reduction in the frequency of alarms in the ICU minimizes alarm fatigue (Blum and Tremper 2010).

A direct comparison of these results with the performance of MEWS reported in previous studies may not be feasible since the datasets, the frequency of measurements, and the outcomes analyzed vary among MEWS studies. Noticeably, studies that evaluated the effect of MEWS implementation on cardiac and cardiopulmonary arrests reported mixed performance results (Subbe *et al* 2003, Jones *et al* 2011, Moon *et al* 2011, Churpek *et al* 2012). It’s worth emphasizing that MEWS was not designed to be a predictor of code blue events nor it is the standard of care to use MEWS in ICU settings. It’s a screening tool to identify inpatients at risk for deterioration and to trigger early evaluation and transfer to step-down or intensive care. The low precision and high false positive rate in ICU population we analyzed do not necessarily indicate a deficiency of MEWS. We simply used MEWS as un exemplar of a clinical prediction index to examine characterization and performance evaluation techniques, and not to examine the performance of MEWS as a predictor of code blue events.

By adopting an event-level evaluation of MEWS where only alarms triggered within a defined nursing shift period of 12 h are considered true alarms, performance levels changed significantly compared to those obtained under patient-level evaluation. Notably, the number of workups required to detect a cardiac arrest dropped significantly by close to 50%, from 10.7 to 5.3 (see section 3.1). Less patients to evaluate translates to less disruption of the clinical workflow and care cost saving. This improvement in performance is due to reducing the probability of false positives by limiting false alarms to those occurring within nursing shifts in a control recording as defined in the event-level evaluation (see section 2.3.3.2). Many control patients may have sporadic false alarms, which under the proposed event-level evaluation are less likely to trigger randomly sampled windows and lead to false positives. It is important to note that the proposed definition of WDR does not consider the burden of alerts leading to a workup. One may define an alarm-level WDR that measures how many false alarms get triggered before a cardiac arrest gets detected (true alarm), which can be calculated as the total number of true and false alarms to the number of true alarms. An efficient predictive system is one that demonstrates a practically low level of alarms per outcome.

The prediction horizon is a parameter that can be selected based on the clinical relevance of an application. The choice of 12 h nursing shift as a prediction horizon for the evaluation of MEWS in this study was motivated by the need to select an actionable timeframe within which MEWS alerts should lead to intervention to demonstrate the proposed performance metrics. We used 12 h here because it is the typical duration of a nursing shift in US hospital settings, including critical care (Stimpfel *et al* 2019). In an ICU unit, two teams of nurses—one per shift—ensure around the clock monitoring. Assuming MEWS threshold is adjustable by the attending nurse, MEWS alerts could be evaluated relative to the nursing shift where they were triggered. A nurse covering the morning shift may set the threshold to different value than the nurse from the previous night shift.

Interestingly, conventional MEWS led to similar sensitivity as with MEWS calculated from worst case values (see section 3.2). The alarm burden, however, was less with MEWS_worst_ than with conventional MEWS (see section 3.3). Despite a slightly lower proportion of on-time alarms and higher missed alarms, MEWS_worst_ may be regarded as better choice than conventional MEWS as it maintains a high level of sensitivity and alleviates the alarm burden. The probability distribution of generating alarms did not substantially differ between MEWS implementations tested here, indicating that different MEWS calculation methods change the time distribution of alerts but not their predictive power.

## 5. Conclusion

Comprehensive evaluation of predictive alarm algorithms to establish their true predictive power and clinical usefulness is lacking. This study proposes approaches and technical considerations to quantify the performance and practicality of predictive alarm algorithms in predicting adverse clinical events. Using MEWS as a case study, revisited classic measures of performance and tools that capture and illustrate the burden of alarms are presented and compared to conventional approaches. The proposed approach addresses the incompleteness and limitation of classic measures to incorporate key clinical considerations and suggests measures and methods that capture the clinical burden and the timeliness of alarms to guide the judgment about the practicality and utility of a candidate predictive alarm algorithm.

Work performed at the Department of Physiological Nursing, School of Nursing, UCSF, San Francisco, CA and at the Office of Science and Engineering Laboratories, Center for Devices and Radiological Health, Food and Drug Administration, Silver Spring, MD.

## Conflicts of interest and source of funding

None declared

Reprints will not be ordered.

## Funding

This work was supported in part by the National Institutes of Health (grant R01HL128679) and by the Center of Excellence in Regulatory Science and Innovation (CERSI) grant to University of California, San Francisco (UCSF) and Stanford University from the US Food and Drug Administration (U01FD005978). Its contents are solely the responsibility of the authors and do not necessarily represent the official views of the HHS or FDA.
